# Epstein–Barr virus is associated with periodontal diseases: A meta-analysis based on 21 case–control studies

**DOI:** 10.1097/MD.0000000000005980

**Published:** 2017-02-10

**Authors:** Zilong Gao, Juan Lv, Min Wang

**Affiliations:** a Department of Oral and Maxillofacial Surgery, Dongfeng Stomatology Hospital. The Genetal Hospital of Dongfeng, Hubei University of Medicine, Shiyan, China; b Department of Oral Implantology, School and Hospital of Stomatology, Wuhan University, Wuhan, China; c Department of Stomatology, HuaGuo Hospital of Dongfeng Auto Corporation, Shiyan, China; d Department of Stomatology, Zhongnan Hospital of Wuhan University, Wuhan, China.

**Keywords:** EBV, Meta-analysis, periodontal diseases

## Abstract

Some controversies still exist between the detection of Epstein–Barr virus (EBV)'s DNA and risks of periodontal diseases. Hence, a comprehensive meta-analysis on all available literatures was performed to clarify the relationship between EBV and preidontitis.

A comprehensive search was conducted within the PUBMED, EMBASE, and WANFANG databases up to October 10th, 2016 according to inclusion and exclusion criteria and finally 21 case–control literatures were obtained. The outcomes including odds ratios (ORs) with 95% confidence intervals (CIs) were used to assess the strength of associations. Publication bias was determined by Begg or Egger test. Sensitivity analysis was used to investigate reliability and stability of the results.

According to the data from included trials, the association between overall increased risks of periodontitis and the detection of EBV was significant (OR = 6.199, 95% CI = 3.119–12.319, *P* < 0.001). In the disease-type analysis, the pooled ORs for chronic periodontitis and aggressive periodontitis were 6.586 (95% CI = 3.042–14.262, *P* < 0.001) and 8.361 (95% CI = 2.109–33.143, *P* = 0.003), respectively. In the subgroup analysis of ethnicity, our results suggested that high EBV-detecting frequencies were correlated with increased risks of periodontitis in Asians, Europeans, and Americans (*P* < 0.001). Subgroup analysis by the sample type showed that subgingival plaque (SgP) samples and tissue samples were available for EBV detecting (*P* < 0.001). Detecting EBV of samples in ≥5 (6) mm sites of periodontal pockets were easier than in ≤3-mm sites (*P* = 0.023).

This meta-analysis indicates that high frequent detection of EBV correlates with increased risk of periodontal diseases. SgP and tissue are available for detecting EBV in patients of periodontitis. At last, our results suggest that detecting EBV of samples in =5 (6) mm sites of periodontal pockets are more sensitive than in ≤3-mm sites.

## 1 Introduction

Periodontitis is a chronic inflammatory disease that is characterized by periodontal damage, alveolar bone resorption, pain, and eventual tooth loss.^[1]^ The pathogenesis of periodontitis is considered to involve complex interactions between microbial factors, host factors, and a variety of environmental factors.^[1]^ The subgingival plaque is required for the initiation of the disease.^[2]^ Interestingly, several studies suggested that the current theory of bacterial plaque cannot explain that patients who are absent of these specific bacterial species still got periodontal diseases.^[3,4]^ And no significant difference in the prevalence of bacteria between healthy and diseased periodontal tissues has been found.^[5]^ Therefore, human herpesviruses have been found to be involved in the etiology of periodontitis because bacterial activity alone is not able to explain all the clinical characteristics of periodontal diseases.^[6]^

EBV, also called human herpesviruses 4 (HHV-4), belongs to γ-herpes virus subfamilies. EBV has widely infected >90% adults in the world and is associated with many human diseases, such as post-transplate lymphoproliferative diseases, nasopharyngeal carcinoma, and oral hairy leukoplakia.^[7]^ The first report about the relationship between EBV and chronic periodontitis (CP) came into our sight in 1996.^[8]^ Afterwards, a number of articles^[9–29]^ have investigated the associations between EBV and periodontal diseases including CP and aggressive periodontitis (AgP). However, these findings were full of controversy among detecting EBV existence in the periodontal environment. Some studies^[10–12,14,15,17–23,26–29]^ have reported that with high prevalence of EBV DNA detecting, the risks of periodontal diseases are significantly increased; whereas others^[9,13,16,24,25]^ suggested a weak or even no relationship between them. Hence, we performed the current comprehensive meta-analysis, which combines results from literatures to confirm whether the EBV is associated with periodontal diseases.

## 2 Materials and methods

### 2.1 Literature search

A systematic search was conducted within PubMed, Embase, and Wanfang databases up to October 10th, 2016 by ZG and MW, using the following key terms: “EBV” and “periodontitis OR periodontal disease.” Reference lists of selected literatures were examined manually for other available publications.

### 2.2 Inclusion and exclusion criteria

The studies were included with the following inclusion criteria: the experimental design was a case–control study; the topic estimated the association between periodontal diseases and EBV; (iii) sample-extracting methods were limited as following: surgery, paper point, curette, paper strip, and biosy; (vi) the patients and controls must be systemically healthy; (v) articles must offer the sample sizes, odds ratios (ORs), and its 95% confidence intervals (CI) or sufficient data to evaluate the association between periodontal diseases and EBV. The major exclusion criteria were as follows: no case–control studies; participants with systemic disease; saliva samples; (vi) no useful data could be extracted or obtained; (v) diseases are not diagnosed as periodontitis or periodontitis-similar diseases.

### 2.3 Data extraction

The following information was selected independently by 2 authors (ZG and JL) according to the criteria listed previously: the first author's name, publication year, country, sample size, sample type, sampling method, and disease type. All controversial questions were resolved by asking a third author. All extracted data were based on previous published studies; thus, no ethical approval and patient consent are required.

### 2.4 Statistical analysis

First, the heterogeneity test was detected with use value. A fixed-effects model was selected if the *P* value of *I*^2^ was <50%; otherwise, the random-effects model was chosen. ORs and the corresponding 95% CIs were conducted to evaluate the association between detection of EBV and periodontal disease risks. Sensitivity analysis was investigated to assess the robust of pooled results by omitting one study each time. The publication bias was determined by the Begg rank correlation test and Egger linear regression test. *P* < 0.05 was considered statistically significant, and all *P* values were 2-sided. The ORs and 95% CIs in this meta-analysis were performed using Stata 12.0 (StataCorp LP, College Station, TX).

## 3 Results

### 3.1 Summary of studies’ characteristics

**Figure 1:**
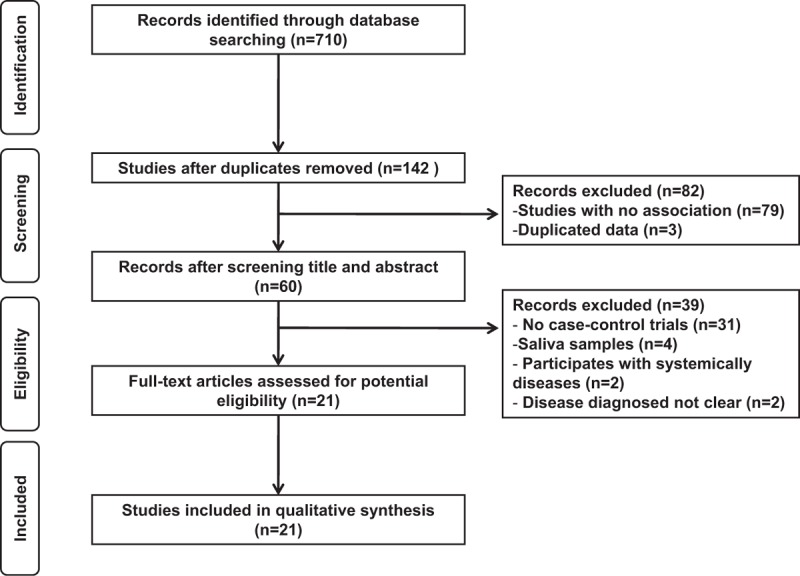
Flow chart of the literature search used in this meta-analysis.

Total 710 potential relevant literatures were collected from the initial search. After duplicates removing with EndNote, 142 studies were remained. Through screening titles and abstracts, 79 irrelevant studies were excluded; the remaining 60 records, which investigated the associations between EBV and different periodontitis, were eligibly evaluated with full-text reading. According to our inclusion criteria, 21 studies,^[9–29]^ including 995 patients and 564 healthy people, were eventually included in our meta-analysis. Figure 1 shows the eligible selecting process.

Among all the selected studies, 15 were conducted in CP patients and 8 were in AgP patients. Probands of 10 studies were in Asian participants, 8 in whites, and 3 in Americans. Sample types included 19 studies in subgingival plaque (SgP) samples, 2 in tissue samples, and 1 in gingival crevicular fluid (GCF) samples. Three studies reported detecting EBV in different depths of periodontal pocket of periodontitis patients. Table 1 shows the main characteristics of the included studies.

**Table 1:**
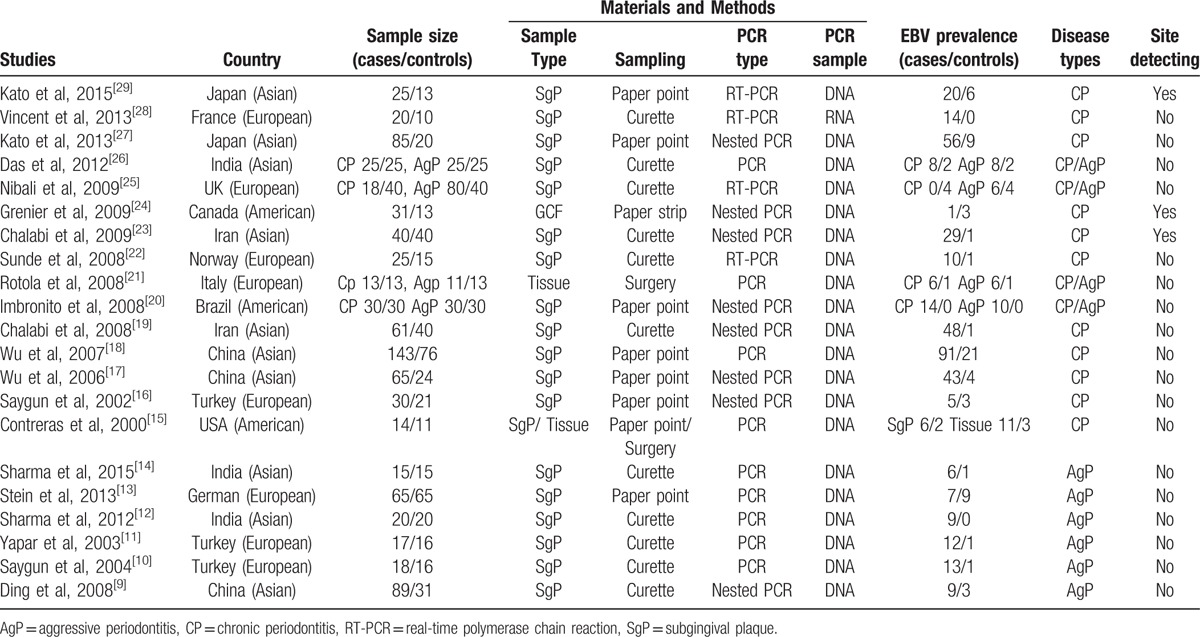
Characteristics of studies included in the meta-analysis.

### 3.2 Overall association of EBV and periodontitis

**Figure 2:**
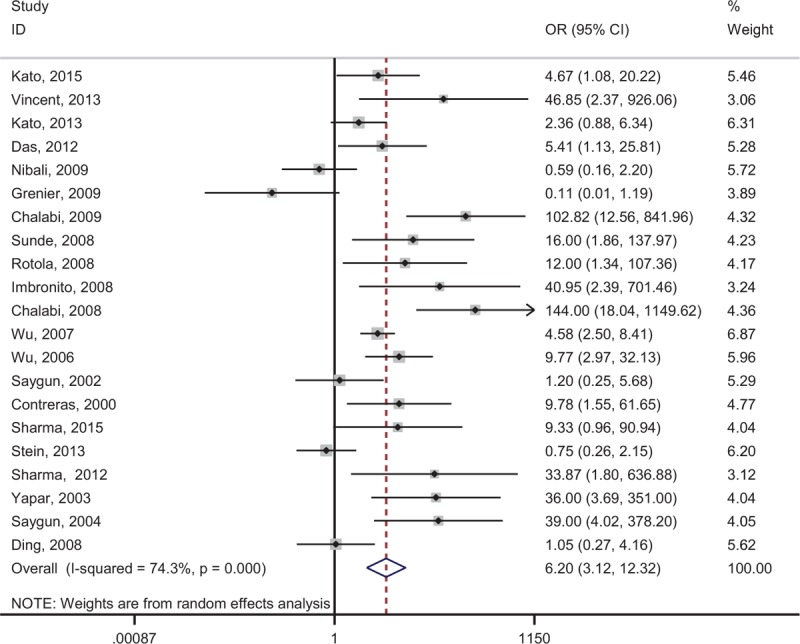
Forest plot of the association between EBV and risk of periodontitis.

To assess the relationship between the risk of periodontal diseases and EBV detecting, all the relative researches (n = 21) containing 995 patients with periodontal diseases and 564 periodontal healthy controls were included. The forest plot is shown in Figure 2 There was significant heterogeneity between these studies (*P* < 0.001, *I*^2^ = 70.5%); a random-effect model was assumed. The pooled results showed that EBV infection is associated with the increased risk of periodontitis (OR = 6.199, 95% CI = 3.119–12.319, *P* < 0.001, Table 2).

**Table 2:**
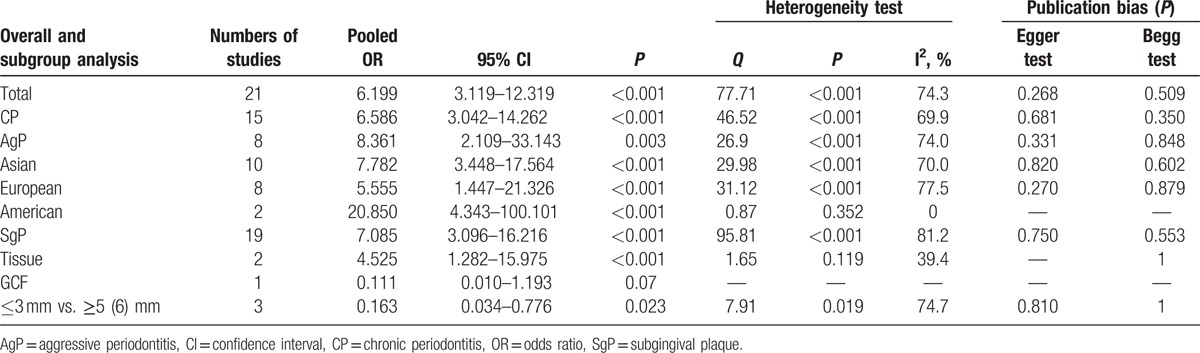
Results including overall and subgroup analysis of pooled OR, 95%CI, *P*, heterogeneity test, and publication bias.

### 3.3 Subgroup analysis

Subgroup analysis by disease types of periodontitis indicated that high detecting frequencies of EBV were significantly associated with increased risks of CP (OR = 6.586, 95% CI = 3.042–14.262, *P* < 0.001) and AgP (OR = 8.361, 95% CI = 2.109–33.143, *P* = 0.003).

Subgroup analysis by origin demonstrated that EBV detections were significantly associated with increased risks of periodontal diseases both in Asians (OR = 7.138, 95% CI = 5.063–10.061, *P* < 0.001), Europeans (OR = 5.555, 95% CI = 1.447–21.326, *P* < 0.001), and Americans (OR = 20.850, 95% CI = 4.343–100.101, *P* < 0.001, Table 2).

Subgroup analysis by sample type suggested that detecting EBV by SgP (OR = 7.085, 95% CI = 3.096–16.216, *P* < 0.001) and tissue sample (OR = 4.525, 95% CI = 1.282–15.975, *P* < 0.001) were associated with periodontitis. However, only 1 study reported that detecting EBV by GCF sample was not associated with CP (OR = 0.111, 95% CI = 0.010–1.193, *P* = 0.07, Table 2). These results indicated that comparing with SgP and tissue sample, detecting GCF sample may not be very sensitive. When comparing of samples from ≤3 mm and ≥5 (6) mm sites in periodontal pockets, results suggested that samples from deep sites were easier to detect EBV DNA in periodontitis patients (OR = 0.163, 95%CI = 0.034–0.776, *p* = 0.023, Table 2).

### 3.4 Sensitivity analysis and Publication bias

One included study of this meta-analysis was omitted each time to evaluate the stability of pooled results. The result of sensitivity analysis indicated that the study published by Grenier et al^[24]^ in American subgroup analysis was not stable and affected the pooled OR (OR = 3.435, 95% CI = 0.122–96.836, *P* = 0.469). After excluding this study by Grenier et al,^[24]^ the meta-analysis of remaining studies become stable (OR = 20.850, 95% CI = 44.343–100.101, *P* < 0.001). The results of sensitivity analysis for other meta-analysis were remained similar after excluding 1 study each time.

Begg test and Egger test were used to evaluate the publication bias; the results were summarized in Table 2. No significant publication bias was observed in this meta-analysis.

## 4 Discussion

Periodontitis is a chronic disease that affects the majority of adults around the world.^[1]^ Although a variety of presume bacteria are considered to be indispensable in the initiation of periodontitis, it is hard to explain the following clinical characteristics: rapid attachment loss and bone destruction with minimal plaque, special site in periodontitis, and existences of disease activity and quiescence phases.^[6,30]^ So more and more studies have paid attention to the relationship between herpes viruses and different types of periodontitis.^[9–29]^ In this study, we focused on whether EBV, a kind of γ-herpes viruses, has an association with increased risks of periodontal diseases.

Numerous studies have reported that herpes viruses, especially EBV and human cytomegalovirus have significant associations with increased risks of varieties of periodontitis, such as CP and AgP.^[10–12,14,15,17–23,26–29]^ However, there are still some controversies in these findings. Some literatures indicated that a weak or even no relationship exists between herpes viruses and risks of periodontitis.^[9,13,16,24,25]^ A meta-analysis has summarized the relationships between risks of CP and herpes viruses.^[31]^ In this study, we made a comprehensive meta-analysis to clarify the associations of EBV and periodontal diseases based on existing research data.

First, we have searched all the literatures relevant to EBV and periodontal diseases and synthesized data from selected studies. The results have shown a significant association between EBV and periodontitis (Table 2). Then, the result of subgroup analysis indicated that EBV-detecting frequencies were associated with increased risks of CP (Table 2), which was similar to the previous study conducted by Zhu et al.^[31]^ In addition, we, for the first time, made a meta-analysis to demonstrate that EBV was associated with the increased risk of AgP (Table 2). By using a generalized linear mixed model with a logit link, Dawson et al analyzed the correlation between EBV and probing depth (PD), clinical attachment loss (AL), and bleeding on probing (BOP). Results showed that the relationship of liner only exists between EBV and BOP.^[32]^ Wu et al have found the similar conclusion.^[17,18]^ Although nonlinear, the relationship between EBV and PD determined by receiver-operator curve analysis is significant.^[32]^

Subgroup analysis conducted according to ethnicity indicated that EBV was associated with periodontal diseases in Asian (Table 2), European (Table 2), and American (Table 2). Separated subgroup analysis discriminated by the sample type identified significant associations between increased risks of periodontal diseases and detecting EBV in SgP (Table 2) and tissue sample (Table 2), but not in GCF (Table 2), indicating that possible sensitive samples were SgP and tissues. Imbronito et al^[33]^ found that detecting EBV in saliva sample had only a 22% sensitivity rate when comparing with that in SgP sample. Moreover, comparing of sampling sites in ≤3 mm with =5 (6) mm from periodontal pockets of patients, our result suggested that it was easier to detect EBV in deep sites than shallow sites (Table 2).

We did not include the studies evaluating the relationship between other herpes viruses and periodontitis because the main purpose of this meta-analysis is to assess the association between detecting of EBV as a single risk factor and the increased risk of periodontal diseases. Vincent et al^[34]^ found that EBV already exists in epithelial cells of periodontium (pECs) before the initiation of periodontitis, and the extent of EBV in pECs is increased with periodontitis severity. Similar results were described by Kato et al.^[29]^ The ORs of co-infection between EBV and *Porphyromonas gingivalis* were also higher in patients with CP than in health donators.^[29,35]^ Interestingly, Saygun et al^[36]^ found that the EBV has a correlation with *P gingivalis* but not *A actinomycetemcomitans* in AgP. Above all, no matter in CP or in AgP, the co-infection between EBV and *P gingivalis* may be a kind of fixed group of periodontopathic microorganisms. Studies suggested that EBV infection has an association with a median 1.5 times further increase of the high cytokine TNF-α expression, inflammatory processes of bone destruction and clinic symptomatic expressions in periapical lesions.^[37]^ Other studies included in our meta-analysis only evaluated the relationships between EBV and periodontal diseases.

The results of this meta-analysis also showed that EBV had severe associations with significant increased risks of different periodontal diseases, and these results may provide a new treatment strategy to substitute or assist traditional ways for periodontal disease treating, especially for late-stage disease. Some study groups tried to use physical or drug therapy to treat patients with EBV-positive periodontal diseases and found it was useful. Martelli et al^[38]^ found that ND:YAG laser therapy is effective against EBV-positive periodontal diseases. A patient with refractory periodontitis and high EBV levels has significantly improved disease situation after receiving valacyclovir hydrochloride treatment.^[39]^ Most of the time, EBV is well controlled by human's immune system and is restricted in the status of latent infection. In people with immunodeficiency or some other specific situations, the latent infection of EBV will turn into lytic infection. The massive viral replication occurs, which leads to the death of host cells. The virion would be released into extracellular secreta, such as saliva.^[41]^ Grande et al^[40]^. indicated that detections of EBV were more frequently in saliva and in subgingival plaque of HIV-positive patients than in HIV-negative periodontitis patients. However, no evidence has demonstrated whether lytic infection of EBV finally causes periodontitis, or periodontitis cause lytic infection happening. Therefore, more clinical application studies and further molecular mechanism researches are needed to determine the real relationships between EBV and periodontal diseases.

There are several limitations to current meta-analysis. First, the results of our meta-analysis were only applicable for CP and AgP because the associations between EBV and other types of periodontal diseases have not been reported. Second, it is possible to lead a language bias because all the inclusive studies were published in English and Chinese. Third, parts of subgroup analysis in this meta-analysis such as tissue, GCF samples, and site detecting included less studies, which may not provide a strong evidence to support the final results. Fourth, all the studies only referred to 3 ethnic groups—Europeans, Americans, and Asians, which may lead our meta-analysis limitative in application. Last, significant heterogeneity was detected between the studies included in quantitative synthesis. Through further subgroup analysis, we still cannot find the origin of heterogeneity. Hence, our results should be treated as exploratory and with caution.

## 5 Conclusion

Our meta-analysis based on 21 studies including 995 patients of periodontal diseases and 564 healthy people suggests that EBV is associated with increased risks of periodontitis including CP and AgP. In addition, this relationship exists in Asians, Europeans, and Americans. SgP and tissue are available for detecting EBV in patients of periodontitis. However, because of lack of enough evidence, detecting EBV in GCF sample still remains uncertain. At last, our results suggest that detecting EBV in samples from ≥5 (6) mm sites of periodontal pockets are more sensitive than ≤3-mm sites.
